# Hepatic arterial interventional therapies alone or in combination with molecular targeted therapies and PD-(L)1 inhibitors in locally aggressive, early recurrent hepatocellular carcinoma: a retrospective study

**DOI:** 10.3389/fimmu.2025.1643082

**Published:** 2025-09-12

**Authors:** Weixin Luo, Lixuan Liu, Wenping Lin, Jie Mei, Yansong Lin, Zhoutian Yang, Fangyi Liu, Wei Wei, Rongping Guo, Jingping Yun

**Affiliations:** ^1^ State Key Laboratory of Oncology in South China, Guangdong Provincial Clinical Research Center for Cancer, Sun Yat-Sen University Cancer Center, Guangzhou, China; ^2^ Department of Pathology, Sun Yat-Sen University Cancer Center, Guangzhou, Guangdong, China; ^3^ Department of Liver Surgery, Sun Yat-Sen University Cancer Center, Guangzhou, Guangdong, China

**Keywords:** hepatocellular carcinoma, early recurrence, Milan criteria, immune checkpoint inhibitor, hepatic arterial interventional therapy, molecular targeted therapy

## Abstract

**Background:**

Current treatment strategies for locally aggressive (beyond Milan criteria), early recurrent hepatocellular carcinoma (erHCC) lack consensus. This study aims to compare the efficacy of hepatic arterial interventional therapies (HAIT) combined with molecular targeted therapies and PD-(L)1 inhibitors (HAIT-M-P) versus HAIT alone for locally aggressive erHCC.

**Methods:**

This study retrospectively reviewed the data of locally aggressive erHCC patients treated with HAIT alone or HAIT-M-P at Sun Yat-sen University Cancer Center from 2020 to 2024. The progression-free survival (PFS), overall survival (OS), tumor responses, and treatment-related adverse events (TRAEs) were compared. Propensity score matching (PSM) and multivariate Cox regression model were used to minimize confounding bias.

**Results:**

A total of 101 patients with locally aggressive erHCC were enrolled. Compared with the HAIT group (n=51), the HAIT-M-P group (n=50) demonstrated significantly longer median PFS (10.1 months vs. 3.7 months, HR = 0.36, *P* < 0.001) and comparable median OS (not reached vs. 38.2 months, HR = 0.45, *P* = 0.065). After PSM, 24 pairs of patients were included. The HAIT-M-P group maintained a significant median PFS advantage (12.8 months vs. 3.7 months, HR = 0.28, *P* < 0.001) and comparable median OS (not reached vs. 38.2 months, HR = 0.56, *P* = 0.330). In the multivariate Cox regression analysis, the HAIT-M-P group demonstrated a significant improvement in OS (HR = 0.30, *P* = 0.033). The objective response rate and disease control rate were significantly higher in the HAIT-M-P group than in the HAIT group, respectively, according to the RECIST v1.1 (30.0% vs. 7.8%, *P* = 0.009; 82.0% vs. 54.9%, *P* = 0.007) and mRECIST criteria (56.0% vs. 19.6%, *P* < 0.001; 90.0% vs. 58.8%, *P* = 0.001). The grade 3 – 4 TRAEs between the two groups were comparable (19.6% vs. 34.0%, *P* = 0.159).

**Conclusion:**

Compared with HAIT alone, HAIT-M-P was associated with improved PFS and tumor response rates, and showed a possible trend toward improved OS in patients with locally aggressive erHCC, which warrants further validation.

## Introduction

Primary liver cancer is one of the most prevalent malignancies globally and ranks as the third leading cause of cancer-related mortality (1). Hepatocellular carcinoma (HCC), the predominant histological subtype, constitutes 75%–85% of reported cases (1). HCC is characterized by high malignancy, with a 5-year recurrence rate approaching 70% even after curative resection (2, 3).

Recurrent HCC (rHCC) is typically classified into early recurrence (≤2 years) and late recurrence (>2 years) according to the timing of relapse (4, 5). Previous studies have consistently demonstrated that compared to late recurrence, early recurrence exhibits more aggressive features, including higher serum AFP levels, larger tumor diameters, higher risk of extrahepatic spread, and a higher proportion with advanced stage, resulting in significantly worse post-recurrence survival (4, 6, 7). Aggressive recurrence in HCC, a concept recently introduced and defined as recurrence beyond Milan criteria, is associated with a poorer prognosis compared to recurrence within the criteria (8, 9). Among patients with early recurrence, approximately 60% exhibit an aggressive pattern (7), which likely represents one of the worst prognostic subgroups in the rHCC population. Aggressive early-recurrence HCC (erHCC) poses a serious therapeutic challenge, yet current guidelines lack consensus on its standard management. In clinical practice, locally aggressive erHCC is usually treated with hepatic arterial interventional therapies (HAIT, including transarterial chemoembolization (TACE), hepatic arterial infusion chemotherapy (HAIC), and TACE plus HAIC combination therapy) and/or systemic therapy (e.g., molecular targeted therapy or immunotherapy). Although combining HAIT with molecular targeted therapies plus immunotherapies may theoretically outperform HAIT alone for these patients, conclusive clinical evidence is still lacking (10, 11).

Prospective clinical trials EMERALD - 1 (12) and LEAP - 012 (13) showed that in locally unresectable HCC, combining TACE with molecular targeted therapies and PD-(L)1 inhibitors significantly improved progression-free survival compared to TACE plus placebo (EMERALD - 1: 15.0 vs. 8.2 months, HR 0.77, 95% CI 0.61 – 0.98, *P* = 0.032; LEAP - 012: 14.6 vs. 10.0 months, HR 0.66, 95% CI 0.51 – 0.84, *P* < 0.001). Additionally, Chinese multicenter retrospective studies (CHANCE001 and CHANCE2201) suggested that triple therapy (TACE combined with PD-(L)1 inhibitors plus molecular targeted therapies) provided longer overall survival than TACE alone or PD-(L)1 inhibitors plus molecular targeted therapies in unresectable HCC (14, 15). Based on these findings, our study aims to evaluate the survival outcomes and treatment responses between HAIT alone and HAIT in combination with molecular targeted therapies and PD-(L)1 inhibitors as first-line therapy for locally aggressive erHCC.

## Materials and methods

### Patients

We retrospectively reviewed the electronic medical records of HCC patients at Sun Yat-sen University Cancer Center (SYSUCC) who underwent curative surgical resection between January 2020 and December 2023, experienced their initial recurrence within 2 years post-surgery, and subsequently received HAIT alone or combined with molecular targeted therapies and PD-(L)1 inhibitors between April 2020 and July 2024. The inclusion criteria for the study were: (1) pathologically diagnosed with HCC after surgery; (2) older than 18 years; (3) Child-Pugh classification grade A or B; (4) initial recurrence within 2 years post-surgery; (5) exceeding Milan criteria at initial recurrence; (6) at least one measurable intrahepatic lesion; (7) Eastern Cooperative Oncology Group Performance Status (ECOG PS) of 0 – 1; (8) first-line therapy for initial recurrence consisted of HAIT combined with or without PD-(L)1 inhibitors and molecular targeted therapies. The exclusion criteria were as follows: (1) concurrent diagnosis of other malignancies during treatment; (2) a history of esophageal or gastric variceal bleeding; (3) extrahepatic metastasis detected at initial recurrence diagnosis; (4) received fewer than 2 cycles of PD-(L)1 inhibitors in the HAIT combined with molecular targeted therapies and PD-(L)1 inhibitors group (16); (5) follow-up duration less than 1 month; (6) lack of essential clinical data.

### Treatment procedures

HAIT included TACE, HAIC, and TACE plus HAIC combination therapy (TACE-HAIC). HAIC was performed using the FOLFOX regimen (including 5-fluorouracil, oxaliplatin, and leucovorin), with detailed protocols referenced from prior studies (17, 18). For TACE, the tumor-feeding arteries were embolized using a chemotherapeutic emulsion composed of epirubicin, lobaplatin, and lipiodol, as described in previous studies (17, 19). TACE-HAIC followed the same protocols as above, but TACE was performed using epirubicin plus lipiodol (20, 21). The frequency of HAIT was determined by clinical need, with intervals of at least 3 – 4 weeks. For patients receiving HAIT combined with PD-(L)1 inhibitors and molecular targeted therapies, PD-(L)1 inhibitors and molecular targeted therapies were initially administered within 3 days before or after HAIT. Molecular targeted therapies included: apatinib, bevacizumab, donafenib, lenvatinib, and regorafenib. PD-(L)1 inhibitors included: atezolizumab, camrelizumab, pembrolizumab, sintilimab, toripalimab, and tislelizumab. Detailed dosing and administration methods for these drugs are provided in **Supplementary Table S1**. The details of the number of patients receiving different combinations of molecular targeted therapies and PD-(L)1 inhibitors are presented in **Supplementary Table S2**.

### Follow-up and assessment

All patients’ baseline data, including medical records and imaging examinations, were collected. During treatment, blood tests and contrast-enhanced computed tomography (CT) or magnetic resonance imaging (MRI) were performed every 6 – 12 weeks to evaluate efficacy and safety. Comprehensive assessments (including complete blood count, blood chemistry, tumor biomarkers, and CT or MRI scans) were conducted every 3 months in the initial 2-year follow-up period. Thereafter, patients were evaluated every 6 months until disease progression was detected.

Progression-free survival (PFS) was defined as the time from treatment initiation to radiologically confirmed progression or death from any cause during the treatment course. Intrahepatic progression includes primary lesion progression, intrahepatic metastasis, vascular invasion, and bile duct invasion. Extrahepatic progression involves distant metastasis (e.g., to the lungs, bones, or lymph nodes), vascular invasion extension beyond the liver (such as inferior vena cava tumor thrombus or right atrial involvement), and direct invasion of extrahepatic organs. Overall survival (OS) was defined as the time from treatment initiation to death due to any cause. Tumor response, including complete response (CR), partial response (PR), stable disease (SD), and progressive disease (PD), was assessed based on the Response Evaluation Criteria in Solid Tumors version 1.1 (RECIST v1.1) and the modified RECIST (mRECIST) criteria (22, 23). The objective response rate (ORR) was defined as the proportion of patients achieving a CR or PR. The disease control rate (DCR) was defined as the proportion of patients achieving a CR, PR, or SD. Safety was assessed by reviewing medical records. Treatment-related adverse events (TRAEs) were graded according to the National Cancer Institute Common Terminology Criteria for Adverse Events, version 5.0 (CTCAE v5.0).

### Statistical analysis

Continuous variables were expressed as median (interquartile range [IQR]) and compared using the Mann-Whitney U test. Categorical variables were presented as counts (percentages) and assessed using Pearson’s χ² test or Fisher’s exact test. PFS and OS were estimated using the Kaplan-Meier method, with differences evaluated by log-rank test. Propensity score matching (PSM) was performed (nearest-neighbor matching method, caliper = 0.1, 1:1 ratio) between the HAIT and HAIT-M-P groups, with matching variables including age, sex, alpha-fetoprotein (AFP), cirrhosis, Child-Pugh grade, tumor number, tumor size, portal vein tumor thrombus (PVTT), and HAIT type. Cox proportional hazards models were used to estimate hazard ratios (HRs) with 95% confidence intervals (CIs). Variables with *P* < 0.1 in univariable analysis were included in multivariable analysis. The differences in restricted mean survival time (dRMST) and 95% CIs were calculated, with the prespecified time point (t*) which is defined as the minimum of the longest observed follow-up times across groups (rounded down to integers) (24). RMST regression models were constructed using the pseudovalue method, incorporating baseline characteristics and clinically relevant factors potentially affecting survival outcomes (25). Statistical significance was set at *P* < 0.05. All analyses were conducted using R (version 4.4.1) or SAS (version 9.4).

## Results

### Patient characteristics

**Figure 1:**
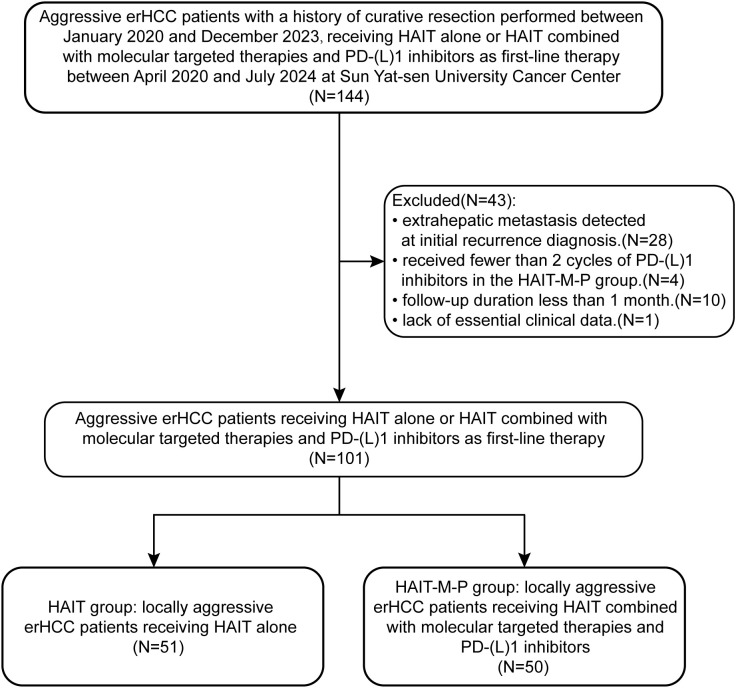
Flow diagram for the patient selection process. erHCC, early recurrent hepatocellular carcinoma; HAIT, hepatic arterial interventional therapy; HAIT-M-P, hepatic arterial interventional therapies combined with molecular targeted therapies and PD-(L)1 inhibitors.

The patient enrollment flowchart is shown in **Figure 1**. A total of 101 patients with locally aggressive erHCC were ultimately included. Among them, 51 received HAIT alone (HAIT group), while 50 received HAIT combined with molecular targeted therapies and PD-(L)1 inhibitors (HAIT-M-P group) as first-line therapy. The baseline characteristics of the enrolled patients are presented in **Table 1**. Our analysis revealed significant differences between the HAIT and HAIT-M-P groups in the distribution of HAIT type and courses of HAIT (*P* < 0.001). After PSM, all variables were comparable between the two groups, as shown in **Table 1**.

**Table 1:** Baseline characteristics of patients in the primary cohort and the propensity score matching cohort.

Variables	Primary cohort			PSM cohort		
	HAIT (N=51)	HAIT-M-P (N=50)	*P*	HAIT (N=24)	HAIT-M-P (N=24)	*P*
Sex			0.714			1.000
male, n (%)	47 (92.2%)	44 (88.0%)		22 (91.7%)	21 (87.5%)	
female, n (%)	4 (7.8%)	6 (12.0%)		2 (8.3%)	3 (12.5%)	
Age (years), median [IQR]	56.00 [50.50, 63.50]	53.00 [47.25, 60.75]	0.284	53.00 [49.75, 58.50]	51.00 [47.00, 59.25]	0.463
Neutrophil (×10^9^/L), median [IQR]	3.01 [2.34, 3.85]	2.80 [2.11, 3.64]	0.292	3.00 [2.34, 3.67]	3.17 [2.72, 3.86]	0.343
Lymphocyte (×10^9^/L), median [IQR]	1.67 [1.40, 2.24]	1.59 [1.35, 2.07]	0.642	1.60 [1.39, 1.90]	1.62 [1.38, 2.41]	0.680
Platelet (×10^9^/L), median [IQR]	166.00 [104.00, 211.00]	162.50 [121.00, 203.00]	0.965	151.50 [94.50, 203.50]	168.50 [127.75, 197.00]	0.284
ALT, U/L			0.775			0.722
≤40, n (%)	39 (76.5%)	36 (72.0%)		20 (83.3%)	18 (75.0%)	
>40, n (%)	12 (23.5%)	14 (28.0%)		4 (16.7%)	6 (25.0%)	
AST, U/L			0.473			1.000
≤40, n (%)	38 (74.5%)	33 (66.0%)		19 (79.2%)	20 (83.3%)	
>40, n (%)	13 (25.5%)	17 (34.0%)		5 (20.8%)	4 (16.7%)	
ALB, g/L			0.986			1.000
≤35, n (%)	1 (2.0%)	2 (4.0%)		1 (4.2%)	0 (0.0%)	
>35, n (%)	50 (98.0%)	48 (96.0%)		23 (95.8%)	24 (100.0%)	
TBil, umol/L			1.000			0.602
≤20.5, n (%)	44 (86.3%)	43 (86.0%)		21 (87.5%)	23 (95.8%)	
>20.5, n (%)	7 (13.7%)	7 (14.0%)		3 (12.5%)	1 (4.2%)	
HBsAg			1.000			1.000
negative, n (%)	8 (15.7%)	7 (14.0%)		4 (16.7%)	4 (16.7%)	
positive, n (%)	43 (84.3%)	43 (86.0%)		20 (83.3%)	20 (83.3%)	
Anti-HCV			1.000			1.000
negative, n (%)	49 (96.1%)	49 (98.0%)		23 (95.8%)	23 (95.8%)	
positive, n (%)	2 (3.9%)	1 (2.0%)		1 (4.2%)	1 (4.2%)	
AFP, ng/mL			0.179			1.000
≤400, n (%)	38 (74.5%)	30 (60.0%)		16 (66.7%)	15 (62.5%)	
>400, n (%)	13 (25.5%)	20 (40.0%)		8 (33.3%)	9 (37.5%)	
Cirrhosis			0.490			0.773
absent, n (%)	18 (35.3%)	22 (44.0%)		13 (54.2%)	11 (45.8%)	
present, n (%)	33 (64.7%)	28 (56.0%)		11 (45.8%)	13 (54.2%)	
Child-Pugh grade			0.121			1.000
A, n (%)	51 (100.0%)	46 (92.0%)		24 (100.0%)	24 (100.0%)	
B, n (%)	0 (0.0%)	4 (8.0%)		0 (0.0%)	0 (0.0%)	
Tumor size, cm			0.337			1.000
≤5, n (%)	47 (92.2%)	42 (84.0%)		21 (87.5%)	21 (87.5%)	
>5, n (%)	4 (7.8%)	8 (16.0%)		3 (12.5%)	3 (12.5%)	
Tumor number			0.484			1.000
single, n (%)	2 (3.9%)	0 (0.0%)		0 (0.0%)	0 (0.0%)	
multiple, n (%)	49 (96.1%)	50 (100.0%)		24 (100.0%)	24 (100.0%)	
PVTT			0.466			0.602
absent, n (%)	48 (94.1%)	44 (88.0%)		23 (95.8%)	21 (87.5%)	
present, n (%)	3 (5.9%)	6 (12.0%)		1 (4.2%)	3 (12.5%)	
HAIT type			<0.001			1.000
HAIC, n (%)	4 (7.8%)	17 (34.0%)		4 (16.7%)	4 (16.7%)	
TACE, n (%)	42 (82.4%)	20 (40.0%)		16 (66.7%)	16 (66.7%)	
TACE-HAIC, n (%)	5 (9.8%)	13 (26.0%)		4 (16.7%)	4 (16.7%)	

PSM, propensity score matching; ALT, Alanine aminotransferase; AST, Aspertate aminotransferase; ALB, albumin; TBil, total bilirubin; HCV, hepatitis C virus; AFP, alpha-fetoprotein; PVTT, portal vein tumor thrombus; HAIT, hepatic arterial interventional therapy; TACE, transarterial chemoembolization; HAIT-M-P, hepatic arterial interventional therapy combined with molecular targeted therapies and PD-(L)1 inhibitors.

### Progression-free survival and overall survival

**Figure 2:**
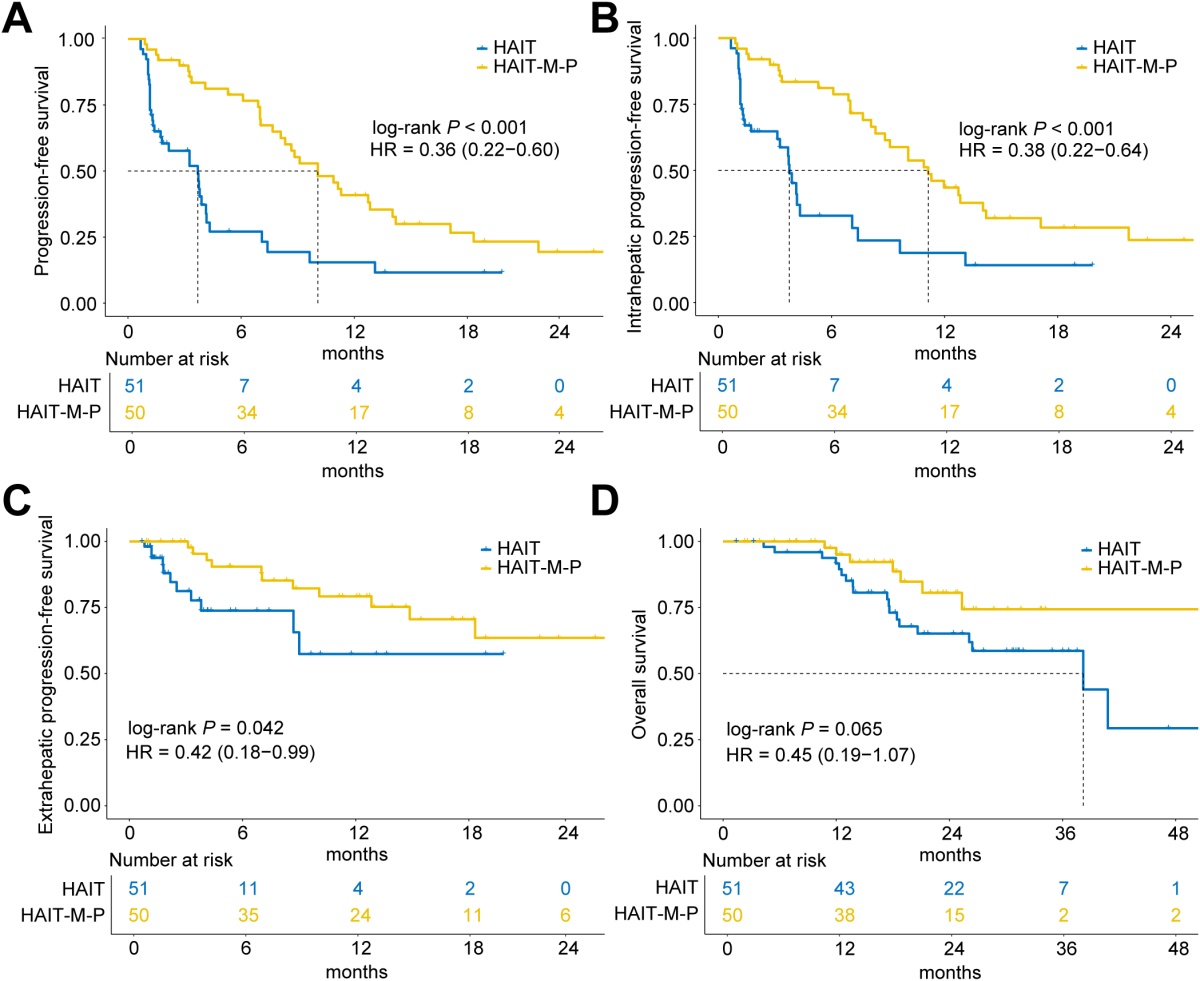
Kaplan-Meier curves of progression-free survival (PFS) and overall survival (OS) for patients in the HAIT (N = 51) and HAIT-M-P (N = 50) groups of the primary cohort. **(A)** PFS, **(B)** intrahepatic PFS, **(C)** extrahepatic PFS, and **(D)** OS of patients in the HAIT and HAIT-M-P groups. HAIT, hepatic arterial interventional therapy; HAIT-M-P, hepatic arterial interventional therapy combined with molecular targeted therapies and PD-(L)1 inhibitors; HR, hazard ratio.

As of January 31, 2025, the median follow-up time for all patients was 23.7 months (IQR, 14.5 – 31.3 months). The HAIT and HAIT-M-P groups had median follow-up durations of 30.1 months (IQR, 18.3 – 31.8 months) and 18.4 months (IQR, 12.1 – 25.6 months), respectively. As shown in **Figure 2A**, the HAIT group exhibited a significantly shorter median PFS of 3.7 months, whereas the HAIT-M-P group demonstrated a markedly longer median PFS of 10.1 months (HR = 0.36, 95% CI: 0.22 – 0.60, *P* < 0.001). When stratified by progression type, the HAIT-M-P group showed superior outcomes in both intrahepatic progression (HR = 0.38, 95% CI: 0.22 – 0.64, *P* < 0.001; **Figure 2B**) and extrahepatic progression (HR = 0.42, 95% CI: 0.18 – 0.99, *P* = 0.042; **Figure 2C**) compared to the HAIT group. Although the HAIT-M-P group showed a trend toward better OS than the HAIT group, the difference did not reach statistical significance (HR = 0.45, 95% CI: 0.19 – 1.07, *P* = 0.065; **Figure 2D**).

**Figure 3:**
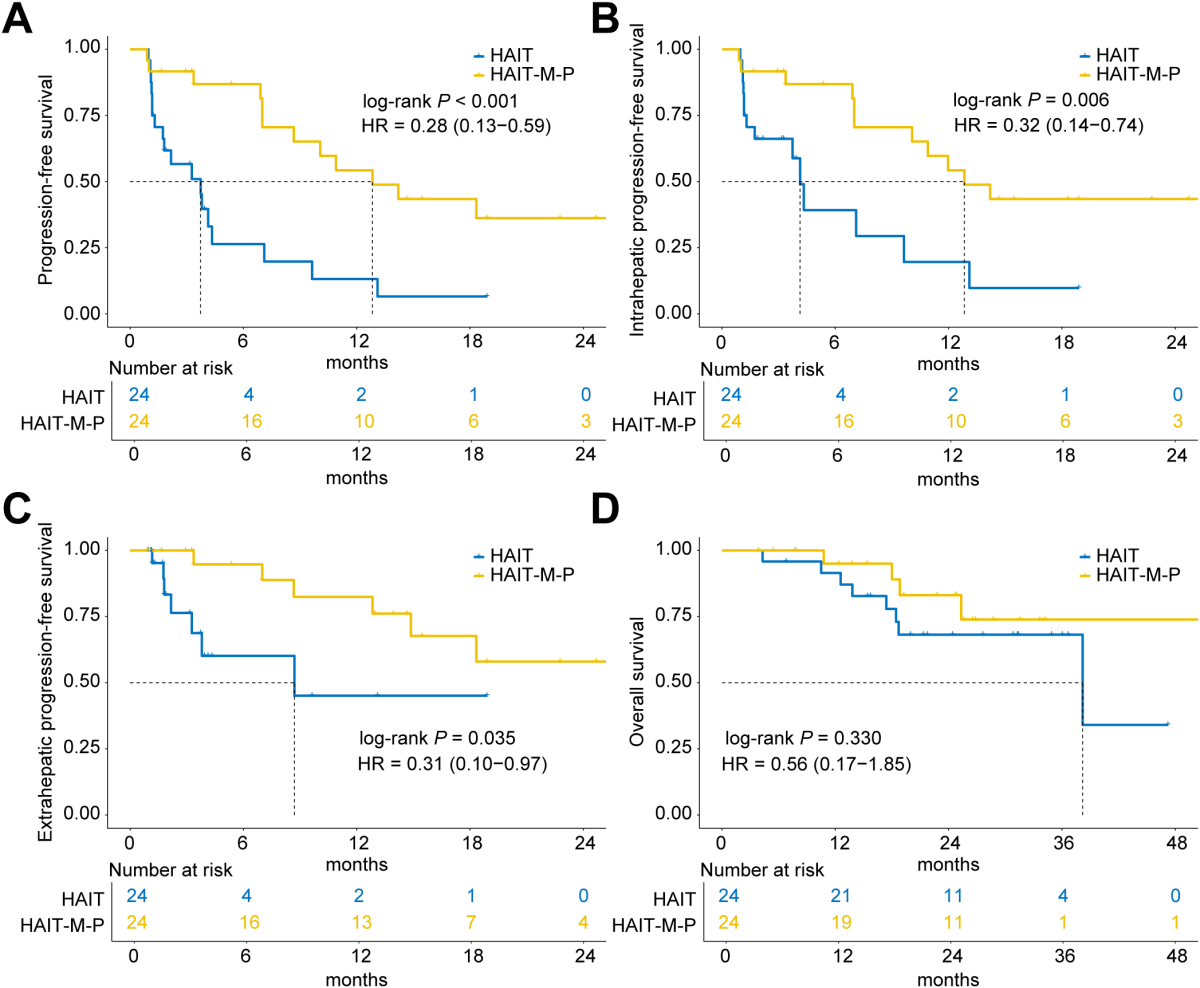
Kaplan-Meier curves of progression-free survival (PFS) and overall survival (OS) for patients in the HAIT (N = 24) and HAIT-M-P (N = 24) groups of the PSM cohort. **(A)** PFS, **(B)** intrahepatic PFS, **(C)** extrahepatic PFS, and **(D)** OS of patients in the HAIT and HAIT-M-P groups. HAIT, hepatic arterial interventional therapy; HAIT-M-P, hepatic arterial interventional therapy combined with molecular targeted therapies and PD-(L)1 inhibitors; HR, hazard ratio.

After 1:1 PSM (24 patients per group), the HAIT-M-P group maintained a significant advantage in overall PFS (median PFS: 12.8 months vs. 3.7 months; HR = 0.28, 95% CI: 0.13 – 0.59, *P* < 0.001; **Figure 3A**). This benefit was consistent for both intrahepatic PFS (HR = 0.32, 95% CI: 0.14 – 0.74, *P* = 0.006; **Figure 3B**) and extrahepatic PFS (HR = 0.31, 95% CI: 0.10 – 0.97, *P* = 0.035; **Figure 3C**). However, OS remained comparable between the two groups (HR = 0.56, 95% CI: 0.17 – 1.85, *P* = 0.330; **Figure 3D**).

As presented in **Table 2**, treatment modality emerged as an independent prognostic factor for PFS, with HAIT-M-P significantly delaying tumor progression compared to HAIT alone. For OS, independent predictors included Child-Pugh grade and treatment modality. Notably, after adjusting for confounding factors, the difference in OS between HAIT-M-P and HAIT reached statistical significance (HR = 0.30, 95% CI: 0.10 – 0.91, *P* = 0.033).

**Table 2:** Univariate and multivariate Cox regression analysis of risk factors for progression-free survival and overall survival in the primary cohort.

Variables	Progression-free survival				Overall survival			
	Univariate		Multivariate		Univariate		Multivariate	
	HR (95% CI)	*p*	HR (95% CI)	*p*	HR (95%CI)	*p*	HR (95% CI)	*p*
Sex (male)	0.87 (0.40 – 1.91)	0.732			1.90 (0.44 – 8.24)	0.390		
Age (≥60 years)	0.58 (0.34 – 0.99)	0.044	0.65 (0.38 – 1.10)	0.106	0.70 (0.28 – 1.74)	0.439		
HBsAg (positive)	1.35 (0.67 – 2.72)	0.401			0.80 (0.27 – 2.33)	0.678		
AFP (>400 ng/mL)	1.02 (0.62 – 1.69)	0.929			1.47 (0.67 – 3.22)	0.339		
Cirrhosis (present)	0.92 (0.57 – 1.49)	0.734			2.25 (0.93 – 5.47)	0.072	1.62 (0.65 – 4.05)	0.303
Child-Pugh grade B	1.39 (0.43 – 4.46)	0.578			6.70 (1.96 – 22.9)	0.002	13.43 (2.84 – 63.59)	0.001
Tumor size (>5 cm)	1.18 (0.60 – 2.31)	0.634			0.74 (0.17 – 3.15)	0.685		
Tumor number (multiple)	1.00 (0.14 – 7.26)	0.999			5.77 (0.75 – 44.18)	0.091	3.87 (0.49 – 30.63)	0.200
PVTT (present)	0.81 (0.38 – 1.69)	0.568			0.75 (0.18 – 3.21)	0.703		
HAIT type (HAIC)	1.20 (0.56 – 2.60)	0.635			0.42 (0.07 – 2.54)	0.345		
HAIT type (TACE)	1.62 (0.83 – 3.17)	0.157			1.70 (0.50 – 5.74)	0.392		
Treatment (HAIT-M-P)	0.36 (0.22 – 0.60)	<0.001	0.39 (0.23 – 0.64)	<0.001	0.45 (0.19 – 1.07)	0.072	0.30 (0.10 – 0.91)	0.033

AFP, alpha-fetoprotein; PVTT, portal vein tumor thrombus; HAIT, hepatic arterial interventional therapy; HAIC, hepatic arterial infusion chemotherapy; TACE, transarterial chemoembolization; HAIT-M-P, hepatic arterial interventional therapy combined with molecular targeted therapies and PD-(L)1 inhibitors; CI, confidence interval.

Given that real-world survival data may violate the proportional hazards assumption (26), potentially reducing the statistical power of Cox regression, we further validated our findings using RMST analysis. As detailed in **Supplementary Table S3**, RMST results corroborated the Cox regression findings, confirming that HAIT-M-P was associated with significantly improved survival outcomes compared to HAIT. Compared to the HAIT group, the HAIT-M-P group showed 5.66 months longer RMST for PFS (*P* < 0.001) and 10.84 months longer RMST for OS (*P* = 0.015). After adjusting for confounders in the RMST regression model, the HAIT-M-P group demonstrated statistically significant benefits in both PFS and OS compared to the HAIT group (**Supplementary Table S4**).

### Tumor response

**Table 3** presents the tumor response outcomes. The HAIT-M-P group achieved a significantly higher ORR than HAIT group. According to mRECIST criteria, the ORR was approximately three times higher in the HAIT-M-P group (56.0% vs. 19.6%, *P* < 0.001), while RECIST v1.1 assessments showed an nearly fourfold difference (30.0% vs. 7.8%, *P* = 0.009). Both RECIST v1.1 and mRECIST assessments demonstrated significantly superior DCR in the HAIT-M-P group compared to the HAIT group: 82.0% vs. 54.9% (*P* = 0.007) by RECIST v1.1 and 90.0% vs. 58.8% (*P* = 0.001) by mRECIST. Notably, the HAIT-M-P group showed a higher incidence of CR, with 10 patients achieving CR according to mRECIST criteria, whereas only 3 CR cases were observed in the HAIT group.

**Table 3:** Tumor response of patients between the HAIT group and the HAIT-M-P group in the primary cohort according to RECIST 1.1 and mRECIST.

Response	RECIST 1.1			mRECIST		
	HAIT (N = 51)	HAIT-M-P (N = 50)	*P*	HAIT (N = 51)	HAIT-M-P (N = 50)	*P*
CR	0 (0.0%)	2 (4.0%)	0.243	3 (5.9%)	10 (20.0%)	0.069
PR	4 (7.8%)	13 (26.0%)	0.030	7 (13.7%)	18 (36.0%)	0.018
SD	24 (47.1%)	26 (52.0%)	0.766	20 (39.2%)	17 (34.0%)	0.736
PD	23 (45.1%)	9 (18.0%)	0.007	21 (41.2%)	5 (10.0%)	0.001
ORR	4 (7.8%)	15 (30.0%)	0.009	10 (19.6%)	28 (56.0%)	<0.001
DCR	28 (54.9%)	41 (82.0%)	0.007	30 (58.8%)	45 (90.0%)	0.001

CR, complete response; PR, partial response; SD, stable disease; PD, progressive disease; ORR, objective response rate; DCR, disease control rate; HAIT, hepatic arterial interventional therapy; HAIT-M-P, hepatic arterial interventional therapy combined with molecular targeted therapies and PD-(L)1 inhibitors.

### Subgroup analysis

**Figure 4:**
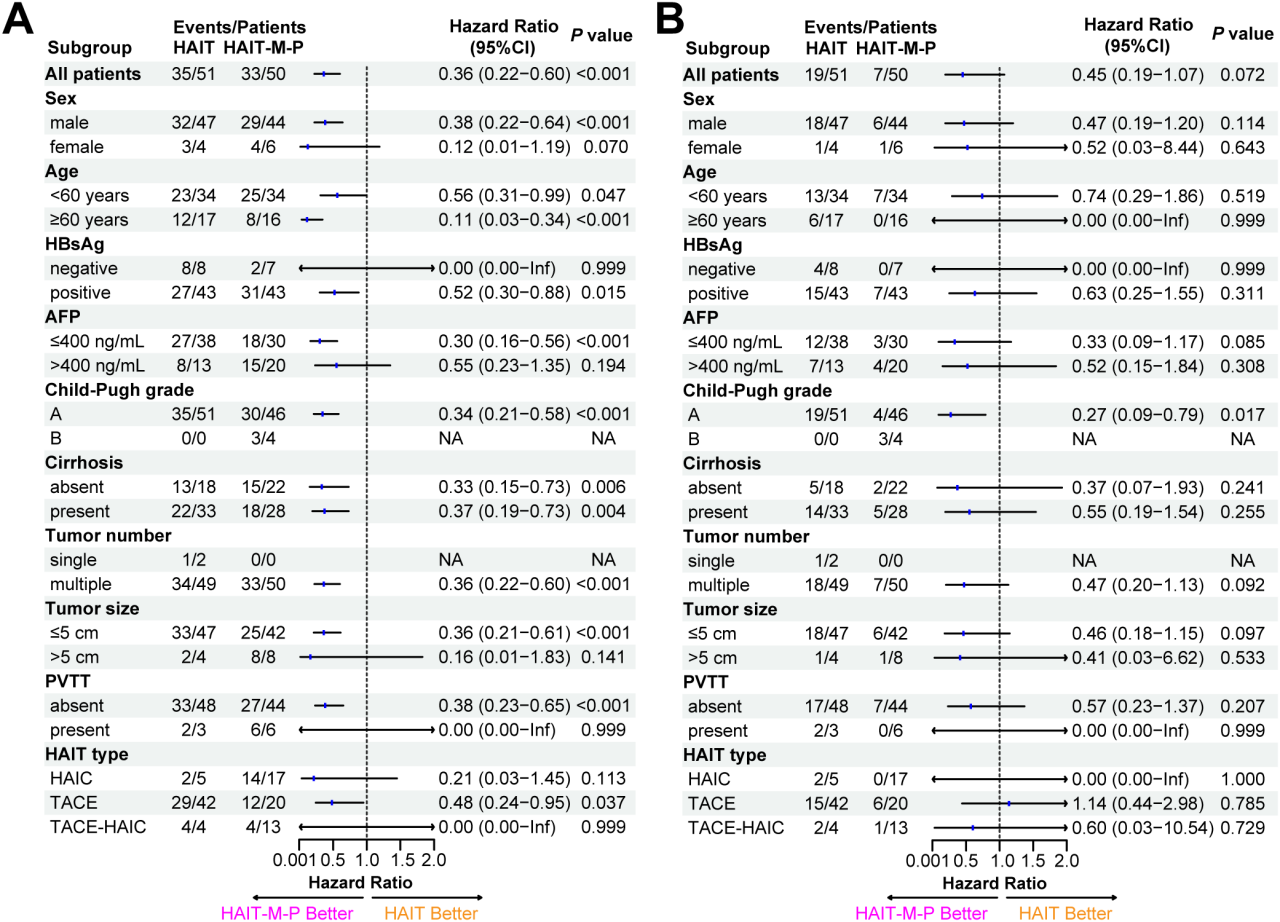
Forest plots of **(A)** progression-free survival and **(B)** overall survival among different patient subgroups in the primary cohort. AFP, alpha-fetoprotein; PVTT, portal vein tumor thrombus; HAIT, hepatic arterial interventional therapy; TACE, transarterial chemoembolization; HAIC, hepatic arterial infusion chemotherapy; HAIT-M-P, hepatic arterial interventional therapy combined with molecular targeted therapies and PD-(L)1 inhibitors; HR, hazard ratio; CI, confidence interval.

The subgroup analysis results for PFS (**Figure 4A**) and OS (**Figure 4B**) are illustrated in the forest plots. Compared to the HAIT group, the HAIT-M-P group demonstrated statistically significant (*P* < 0.05) clinical benefits in PFS among the following subgroups: males, patients aged <60 or ≥60 years, HBsAg-positive individuals, those with AFP ≤400 ng/mL, Child-Pugh grade A, with or without cirrhosis, multiple tumors, tumor size ≤5 cm, absence of PVTT, and those receiving TACE. However, in other subgroups, the effect of HAIT-M-P on PFS remains uncertain due to the limited number of patients (e.g., females, HBsAg-negative individuals, those with tumor size >5 cm, presence of PVTT, and those receiving HAIC or TACE-HAIC), which significantly reduced the statistical power. For OS, the survival benefits were comparable between the HAIT and HAIT-M-P groups in most subgroups. Notably, the HAIT-M-P group had a higher proportion of patients with Child-Pugh grade B. We found that in the Child-Pugh grade A subgroup, the HAIT-M-P group showed a greater OS advantage (HR = 0.27, 95% CI: 0.09 – 0.79, *P* = 0.017).

### Adverse events and safety

As shown in the **Table 4**, overall, the treatment-related adverse events (TRAEs) in the HAIT-M-P group are similar to those in the HAIT group (98.0% vs. 96.1%, *P* = 1.000). For somatosensory TRAEs, fever (14.0% vs. 0.0%, *P* = 0.006) and decreased appetite (36.0% vs. 11.8%, *P* = 0.009) occurred more frequently in the HAIT-M-P group, likely due to cumulative drug effects. More patients in the HAIT-M-P group suffered from neutrophil counts decreased (42.0% vs. 11.8%, *P* = 0.001), total bilirubin increased (68.0% vs. 37.3%, *P* = 0.004), and hypoalbuminemia (42.0% vs. 11.8%, *P* = 0.001) compared with the HAIT group. Despite these differences, the rates of grade 3 – 4 TRAEs were comparable between the two groups (19.6% vs. 34.0%, *P* = 0.159), and no TRAE-related deaths were observed during follow-up. Nearly all TRAEs were effectively controlled with supportive interventions, including anti-allergy therapy, hepatic functional protection, analgesic therapy, and so on.

**Table 4:** Treatment-related adverse events between the HAIT group and the HAIT-M-P group in the primary cohort.

Treatment-related adverse events, n (%)	Any grade			Grade 3/4		
	HAIT (N=51)	HAIT-M-P (N=50)	*P*	HAIT (N=51)	HAIT-M-P (N=50)	*P*
Overall	49 (96.1%)	49 (98.0%)	1.000	10 (19.6%)	17 (34.0%)	0.159
Rash	0 (0.0%)	1 (2.0%)	0.495	0 (0.0%)	0 (0.0%)	NA
Pruritus	0 (0.0%)	2 (4.0%)	0.243	0 (0.0%)	0 (0.0%)	NA
Pain	14 (27.5%)	22 (44.0%)	0.126	3 (5.9%)	1 (2.0%)	0.617
Fever	0 (0.0%)	7 (14.0%)	0.006	0 (0.0%)	0 (0.0%)	NA
Diarrhea	2 (3.9%)	2 (4.0%)	1.000	0 (0.0%)	0 (0.0%)	NA
Fatigue	1 (2.0%)	1 (2.0%)	1.000	0 (0.0%)	0 (0.0%)	NA
Nausea	6 (11.8%)	7 (14.0%)	0.969	0 (0.0%)	0 (0.0%)	NA
Emesis	5 (9.8%)	13 (26.0%)	0.062	0 (0.0%)	0 (0.0%)	NA
Decreased appetite	6 (11.8%)	18 (36.0%)	0.009	0 (0.0%)	0 (0.0%)	NA
Cough	0 (0.0%)	1 (2.0%)	0.495	0 (0.0%)	0 (0.0%)	NA
Edema peripheral	0 (0.0%)	1 (2.0%)	0.495	0 (0.0%)	0 (0.0%)	NA
Alimentary tract hemorrhage	1 (2.0%)	3 (6.0%)	0.362	0 (0.0%)	0 (0.0%)	NA
Hypothyroidism	0 (0.0%)	3 (6.0%)	0.118	0 (0.0%)	0 (0.0%)	NA
Hyperthyroidism	0 (0.0%)	1 (2.0%)	0.495	0 (0.0%)	0 (0.0%)	NA
Hypertension	0 (0.0%)	4 (8.0%)	0.056	0 (0.0%)	0 (0.0%)	NA
Laboratory-related AEs, n (%)						
Anemia	11 (21.6%)	14 (28.0%)	0.604	1 (2.0%)	0 (0.0%)	1.000
Leukopenia	5 (9.8%)	12 (24.0%)	0.101	0 (0.0%)	1 (2.0%)	0.495
Neutrophil count decreased	6 (11.8%)	21 (42.0%)	0.001	0 (0.0%)	1 (2.0%)	0.495
Platelet count decreased	21 (41.2%)	22 (44.0%)	0.932	2 (3.9%)	5 (10.0%)	0.269
Alanine aminotransferase increased	40 (78.4%)	42 (84.0%)	0.645	5 (9.8%)	8 (16.0%)	0.527
Aspartate aminotransferase increased	41 (80.4%)	47 (94.0%)	0.072	5 (9.8%)	11 (22.0%)	0.160
Total bilirubin increased	19 (37.3%)	34 (68.0%)	0.004	0 (0.0%)	4 (8.0%)	0.056
Hypoalbuminemia	6 (11.8%)	21 (42.0%)	0.001	0 (0.0%)	0 (0.0%)	NA
Creatinine increased	10 (19.6%)	6 (12.0%)	0.439	0 (0.0%)	0 (0.0%)	NA

HAIT, hepatic arterial interventional therapy; HAIT-M-P, hepatic arterial interventional therapy combined with molecular targeted therapies and PD-(L)1 inhibitors.

## Discussion

Previous studies have shown that rHCC patients who meet the Milan criteria benefit from radical treatments such as repeat hepatic resection or radiofrequency ablation (27, 28). However, the optimal treatment strategy for HCC patients with aggressive early recurrence (beyond Milan criteria), which likely represents one of the most challenging subgroups within the rHCC population, has yet to be established. In clinical practice, HAIT is extensively applied to locally aggressive rHCC (29–31). Although research has explored HAIC or TACE-HAIC schemes, most studies involve participants with primarily locally unresectable HCC, including only a subset of recurrent cases (21, 32). The studies focusing on TACE for rHCC (it is important to note that these studies vary significantly in their selection criteria) reported a median PFS of only 2.5 – 13.7 months, and a median OS of 15.1 – 24.0 months, which are not satisfactory (31, 33, 34). In recent years, with the widespread use of PD-(L)1 inhibitors, the combination of molecular targeted therapies and PD-(L)1 inhibitors has been recommended as a standard first-line therapy for advanced HCC due to its good effectiveness. A retrospective study suggests that this combination is superior to molecular targeted therapy alone for erHCC (35). Further evidence indicates that a triple therapy regimen combining HAIT with molecular targeted therapies and immune checkpoint inhibitors can provide significant survival benefits (36, 37). Theoretically, for patients with locally aggressive erHCC, the combination of HAIT with molecular targeted therapies plus PD-(L)1 inhibitors may be superior to HAIT alone. However, recent research has shown that erHCC exhibits a greater ability of drug resistance and immune evasion compared to primary tumor and late recurrence (38–40). Besides, recurrence exceeding the Milan criteria indicates a more aggressive tumor biology (8). Thus, the effectiveness of combined treatments in overcoming these challenges remains to be further explored.

This study systematically evaluated the efficacy of HAIT combined with molecular targeted therapies and PD-(L)1 inhibitors versus HAIT alone in patients with locally aggressive erHCC. The results demonstrated that the median PFS in the combination group was significantly extended (10.1 months vs. 3.7 months), and the ORR was nearly tripled (56.0% vs. 19.2%), with benefits maintained after PSM. However, there was no statistically significant difference in OS between the two groups in the primary cohort. Several factors may have contributed to this result. First, the median follow-up duration was shorter in the combination group (18.4 months vs. 27.6 months), which may have limited the ability to observe long-term survival benefits. Second, a considerable proportion of patients in the HAIT alone group received subsequent systemic therapies after disease progression, potentially confounding the OS comparison due to treatment crossover effects. Third, the triple therapy arm had a higher proportion of patients with Child-Pugh B liver function, which could have negatively impacted survival outcomes. Fourth, the relatively small sample size may have limited the statistical power to detect significant differences in OS between groups. After adjusting for confounding factors using the multivariate Cox regression model, the HAIT-M-P group showed a significant improvement in OS compared to the HAIT group (HR = 0.30, *P* = 0.033). The RMST analysis further confirmed this trend, with an extension of OS by 10.84 months (*P* = 0.015) in the HAIT-M-P group. These findings provide an important basis for clinical decision-making for aggressive erHCC.

The observed therapeutic benefits may be attributed to several synergistic mechanisms. First, HAIT induces rapid tumor cytoreduction through local embolization or high-concentration chemotherapy, simultaneously alleviating tumor-mediated immunosuppression and potentially triggering immunogenic cell death (41). Then, molecular targeted therapies directly inhibit tumor proliferation while modulating angiogenesis, thereby improving hypoxia-induced vascular abnormalities, and potentially enhancing the sensitivity to immune checkpoint inhibitors (42). Finally, PD-(L)1 inhibitors may amplify the antigen release induced by HAIT and the microenvironment modulation by targeted therapy, activating systemic anti-tumor immunity (43).

Subgroup analysis suggests that patients who are male, HBsAg-positive, have AFP ≤400 ng/mL, Child-Pugh A, multiple tumors, tumor size ≤5 cm, absence of portal vein thrombosis, and those receiving TACE benefit more significantly from the combination therapy. However, due to the limited sample size in the comparison subgroups, these findings need to be validated in larger-scale studies.

Compared to the HAIT group, the HAIT-M-P group exhibited a higher incidence of fever, decreased appetite, neutropenia, elevated bilirubin, and hypoalbuminemia, which are primarily considered to be related to the cumulative toxicity of molecular targeted therapies and PD-(L)1 inhibitors (44–46). Nonsteroidal anti-inflammatory drugs (NSAIDs) are routinely used during HAIT courses to prevent abdominal pain, and they also have antipyretic effects, which might be one reason why fevers exceeding 38 °C were not observed in the HAIT group. It is important to note that hepatotoxicity is a significant limiting factor in the use of molecular targeted drugs and immune checkpoint inhibitors (47, 48); therefore, the choice of combination therapy should be made cautiously in patients with poor liver function. In this study, there was no significant increase in overall and grade 3 – 4 TRAEs between the two groups, and no treatment-related deaths occurred, suggesting that although the HAIT-M-P group had a higher rate of AEs, they were generally controllable.

There are several limitations in this study. First, it is a single-center, small-sample retrospective study, which may have potential selection bias. However, we have attempted to minimize this bias through various methods such as PSM, Cox multivariate regression analysis, and RMST regression analysis. Additionally, the hepatic arterial interventional therapies included HAIC, TACE, and TACE-HAIC, and there were differences in the molecular targeted therapies and immune checkpoint inhibitors used, which may lead to differences in treatment efficacy. It is necessary to expand the cohort and further explore the optimal combination by subdividing treatment types. Lastly, as this is a retrospective study, although we have carefully reviewed the medical records, some TRAEs that are not directly reflected in the records may not be fully assessed.

In conclusion, our findings suggest that HAIT combined with molecular targeted therapies and PD-(L)1 inhibitors may improve PFS and tumor response rates, and show a trend toward better OS compared to HAIT alone in patients with locally aggressive erHCC. To further validate our findings, studies with larger sample sizes, longer follow-up durations, and prospective cohort designs are needed.

## Data Availability

The original contributions presented in the study are included in the article/**Supplementary Material**. Further inquiries can be directed to the corresponding authors.
