# Supplementary material for: Hepatic arterial interventional therapies alone or in combination with molecular targeted therapies and PD-(L)1 inhibitors in locally aggressive, early recurrent hepatocellular carcinoma: a retrospective study
Source: Front Immunol. 2025 Sep 12;16:1643082. doi: 10.3389/fimmu.2025.1643082 (PMC12463941; doi:10.3389/fimmu.2025.1643082)
Supplement: Supplementary file 1 [file Table1.docx]

**Supplementary Table 1** Summary of PD-(L)1 inhibitors and molecular targeted therapies for locally aggressive early recurrent hepatocellular carcinoma.

| Treatment | Drug name | Administration | Dose | Frequency |
| --- | --- | --- | --- | --- |
| PD-(L)1 inhibitors |  |  |  |  |
|  | Atezolizumab | Intravenous | 1200 mg | Q3W |
|  | Camrelizumab | Intravenous | 3 mg/kg | Q3W |
|  | Pembrolizumab | Intravenous | 200 mg | Q3W |
|  | Sintilimab | Intravenous | 200 mg | Q3W |
|  | Tislelizumab | Intravenous | 200 mg | Q3W |
|  | Toripalimab | Intravenous | 240 mg | Q3W |
| molecular targeted therapies |  |  |  |  |
|  | Apatinib | Oral | 250 mg | QD |
|  | Bevacizumab | Intravenous | 15 mg/kg | Q3W |
|  | Donafenib | Oral | 100 mg | BID |
|  | Lenvatinib | Oral | 8 mg/12mg | QD |
|  | Regorafenib | Oral | 160 mg | QD × 21 d, 28d cycle |
