# Supplementary material for: Hepatic arterial interventional therapies alone or in combination with molecular targeted therapies and PD-(L)1 inhibitors in locally aggressive, early recurrent hepatocellular carcinoma: a retrospective study
Source: Front Immunol. 2025 Sep 12;16:1643082. doi: 10.3389/fimmu.2025.1643082 (PMC12463941; doi:10.3389/fimmu.2025.1643082)
Supplement: Supplementary file 2 [file Table2.docx]

**Supplementary Table 2** Summary of the combination of molecular targeted therapies and PD-(L)1 inhibitors in the hepatic arterial interventional therapies combined with molecular targeted therapies and PD-(L)1 inhibitors (HAIT-M-P) group.

| Combination regimen | N (%) |
| --- | --- |
| Lenvatinib plus Tislelizumab | 9 (18.0%) |
| Bevacizumab plus Atezolizumab | 8 (16.0%) |
| Lenvatinib plus Sintilimab | 8 (16.0%) |
| Bevacizumab plus Sintilimab | 7 (14.0%) |
| Apatinib plus Camrelizumab | 3 (6.0%) |
| Lenvatinib plus Toripalimab | 3 (6.0%) |
| Donafenib plus Atezolizumab | 2 (4.0%) |
| Lenvatinib plus Pembrolizumab | 2 (4.0%) |
| Lenvatinib plus Atezolizumab | 2 (4.0%) |
| Lenvatinib plus Camrelizumab | 2 (4.0%) |
| Donafenib plus Tislelizumab | 1 (2.0%) |
| Donafenib plus Pembrolizumab | 1 (2.0%) |
| Apatinib plus Tislelizumab | 1 (2.0%) |
| Regorafenib plus Sintilimab | 1 (2.0%) |
