# Supplementary material for: Hepatic arterial interventional therapies alone or in combination with molecular targeted therapies and PD-(L)1 inhibitors in locally aggressive, early recurrent hepatocellular carcinoma: a retrospective study
Source: Front Immunol. 2025 Sep 12;16:1643082. doi: 10.3389/fimmu.2025.1643082 (PMC12463941; doi:10.3389/fimmu.2025.1643082)
Supplement: Supplementary file 3 [file Table3.docx]

**Supplementary Table 3** RMST analysis for progression-free survival and overall survival for patients between the HAIT group and the HAIT-M-P group in the primary cohort and the propensity score matching cohort.

| Cohort | Outcome | t^*^ (months) | HAIT  (95%CI) | HAIT-M-P  (95%CI) | dRMST  (95%CI) | *P* |
| --- | --- | --- | --- | --- | --- | --- |
| Primary cohort | | | | | | |
|  | PFS | 19 | 5.29  (3.39–7.19) | 10.95  (9.17–12.73) | 5.66  (3.06–8.27) | <0.001 |
|  | IntraPFS | 19 | 5.99  (3.84–8.15) | 11.43  (9.63–13.23) | 5.44  (2.63–8.24) | <0.001 |
|  | ExtraPFS | 19 | 12.91  (9.91–15.91) | 15.85  (14.16–17.54) | 2.94  (-0.50–6.39) | 0.094 |
|  | OS | 54 | 34.09  (27.76–40.43) | 44.93  (38.91–50.96) | 10.84  (2.10–19.58) | 0.015 |
| PSM cohort | | | | | | |
|  | PFS | 18 | 4.93  (2.70–7.16) | 12.27  (9.71–14.82) | 7.34  (3.95–10.73) | <0.001 |
|  | IntraPFS | 18 | 6.20  (3.36–9.04) | 12.45  (9.92–14.97) | 6.24  (2.44–10.04) | 0.001 |
|  | ExtraPFS | 18 | 10.42  (6.31–14.54) | 15.39  (13.29–17.50) | 4.97  (0.35–9.59) | 0.035 |
|  | OS | 47 | 33.48  (26.45–40.51) | 39.79  (33.59–45.99) | 6.32  (-3.06–15.69) | 0.187 |

t*, the minimum of the longest observed follow-up times across groups, rounded down to integers; dRMST, the difference in restricted mean survival time; dRMST = RMST(HAIT-M-P)-RMST(HAIT); CI, confidence interval; PSM, propensity score matching; PFS, progression-free survival; IntraPFS, intrahepatic progression-free survival; ExtraPFS, extrahepatic progression-free survival; OS, overall survival.
