# Supplementary material for: Hepatic arterial interventional therapies alone or in combination with molecular targeted therapies and PD-(L)1 inhibitors in locally aggressive, early recurrent hepatocellular carcinoma: a retrospective study
Source: Front Immunol. 2025 Sep 12;16:1643082. doi: 10.3389/fimmu.2025.1643082 (PMC12463941; doi:10.3389/fimmu.2025.1643082)
Supplement: Supplementary file 4 [file Table4.docx]

**Supplementary Table 4** RMST regression analysis for progression-free survival and overall survival for patients in the primary cohort.

| Variables | Progression-free survival | |  | Overall survival | |
| --- | --- | --- | --- | --- | --- |
|  | dRMST (95%CI) | *P* |  | dRMST (95%CI) | *P* |
| Sex (male) | 0.41 (-0.84–1.65) | 0.521 |  | 0.28 (-1.71–2.26) | 0.785 |
| Age (≥60 years) | 0.63 (-0.19–1.46) | 0.134 |  | 0.04 (-0.92–1.01) | 0.931 |
| HBsAg (positive) | 0.99 (0.04–1.94) | 0.041 |  | -0.43 (-0.93–0.06) | 0.086 |
| AFP (>400 ng/mL) | -0.37 (-1.44–0.70) | 0.502 |  | -0.85 (-1.94–0.24) | 0.128 |
| Cirrhosis (present) | -0.13 (-1.05–0.78) | 0.776 |  | -0.82 (-1.79–0.14) | 0.094 |
| Child-Pugh grade B | -1.32 (-3.77–1.13) | 0.290 |  | -1.83 (-4.63–0.96) | 0.199 |
| Tumor size (>5 cm) | -0.10 (-1.38–1.19) | 0.880 |  | -0.36 (-1.95–1.23) | 0.656 |
| Tumor number (multiple) | -2.82 (-3.94– -1.70) | <0.001 |  | -0.65 (-2.74–1.44) | 0.542 |
| PVTT (present) | 1.08 (-0.28–2.43) | 0.121 |  | 1.48 (0.25–2.71) | 0.019 |
| HAIT type (HAIC) | -0.28 (-1.54–0.99) | 0.669 |  | -0.74 (-2.03–0.55) | 0.263 |
| HAIT type (TACE) | -0.01 (-1.20–1.18) | 0.989 |  | 0.22 (-0.64–1.07) | 0.617 |
| Treatment (HAIT-M-P) | 2.71 (1.61–3.81) | <0.001 |  | 1.16 (0.12–2.20) | 0.029 |

The minimum of the maximum follow-up times (rounded down to the nearest month) was 7 months for progression-free survival and 17 months for overall survival; dRMST, the difference in restricted mean survival time; AFP, alpha-fetoprotein; PVTT, portal vein tumor thrombus; HAIC, hepatic arterial infusion chemotherapy; TACE, transarterial chemoembolization; HAIT, hepatic arterial interventional therapy; HAIT-M-P, hepatic arterial interventional therapy combined with molecularly targeted treatments and PD-(L)1 inhibitors; CI, confidence interval.
